# Juxtapapillary Choroidal Neovascularization in a Young Woman with Tubulointerstitial Nephritis and Uveitis (TINU) Syndrome with Onset in Pediatric Age

**DOI:** 10.3390/medicina58091260

**Published:** 2022-09-12

**Authors:** Maria Pia Paroli, Daniele Cappiello, Davide Staccini, Anna Clara Tamburrelli, Marino Paroli, Ludovico Iannetti

**Affiliations:** 1 Eye Clinic, Department of Sense Organs, Sapienza University of Rome, Policlinico Umberto I Hospital, Viale del Policlinico 155, 00161 Rome, Italy; 2 Department of Clinical, Anesthesiological and Cardiovascular Sciences, Sapienza University of Rome, ICOT Hospital, 04100 Latina, Italy

**Keywords:** TINU syndrome, diffuse uveitis, optic disk edema, juxtapapillary choroidal neovascularization

## Abstract

We describe the unusual case of a young woman with tubulointerstitial nephritis and uveitis (TINU) with bilateral diffuse uveitis and optic nerve inflammatory involvement since she was a child in the 1990s. Imaging diagnostic tools such as fluorescein angiography, indocyanine green angiography, optical coherence tomography (OCT), and OCT angiography revealed inactive juxtapapillary choroidal neovascularization (CNV) after 25 years of follow-up. After treatment, uveitis went into remission with BCVA 20/20 in both eyes and CNV lesions became inactive. Although anterior uveitis is more frequently reported in TINU, posterior uveitis with inflammatory involvement of the optic nerve should be accurately investigated to rule out juxtapapillary CNV, both at the time of active uveitis and during follow-up, since TINU may be complicated by CNV even at the later stages of the inflammatory process.

## 1. Introduction

Tubulointerstitial nephritis and uveitis syndrome (TINU) is a multi-organ disorder characterized by an association between acute renal injury and ocular inflammation. TINU was reported in the ophthalmological literature for the first time in 1988 by Rosenbaum et al. [1], 13 years after its original description [2] from which the “Dobrin syndrome” name comes. It represents 0.2–2% of the uveitis diagnosis [3,4,5] and approximately 5% of nephritis cases [6]. It is likely underdiagnosed because of a lack of associated signs and symptoms which consequently do not usually lead to targeted investigations [7]. TINU may be related to autoimmune systemic diseases, infections, and previous use of drugs [8]. Although there are reported cases in elderly patients, it typically affects children and young adults, and occurs in females three times more often than in males [5,9]. Clinical features could be non-specific (fever, abdominal pain, loss of weight, tiredness, malaise, anorexia, and headache) and renal involvement [5] could lead to different stages of kidney failure [7]. Uveitis can appear up to more than one year after the episode of nephritis, such as the majority of cases (65%), but can also precede it or be concomitant [8]. Typical ocular involvement is characterized by bilateral anterior uveitis [8], although cases of intermediate and posterior uveitis and panuveitis are not uncommon. Chorioretinal lesions with neovascular membrane are also rarely reported, mainly in adult patients [10]. We describe the case of a TINU diagnosed in childhood, in which the uveitis began as anterior and bilateral at the onset, and was complicated by bilateral papillitis and unilateral juxtapapillary choroidal neovascularization (CNV).

## 2. Case Presentation

**Figure 1:**
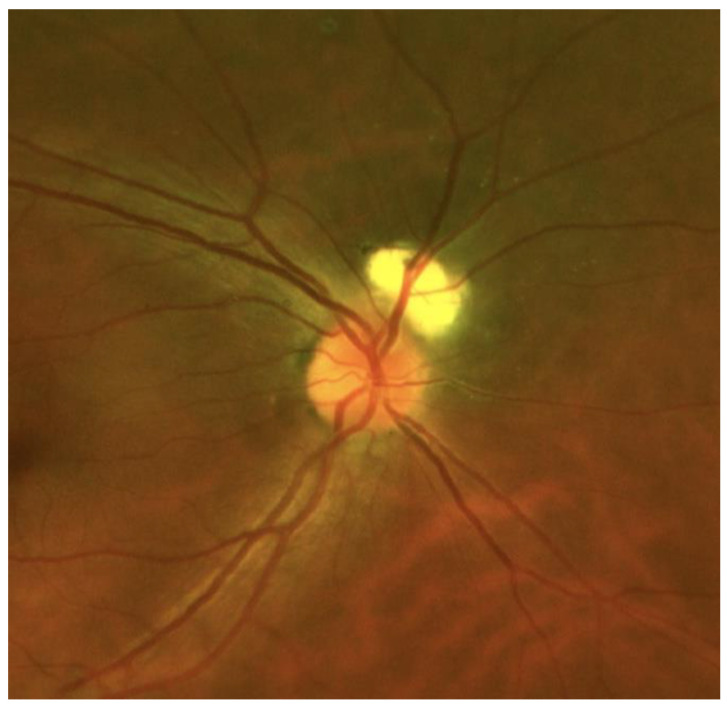
RE wide-field retinography showing a yellowish juxtapapillary superonasal oval shape lesion.

**Figure 2:**
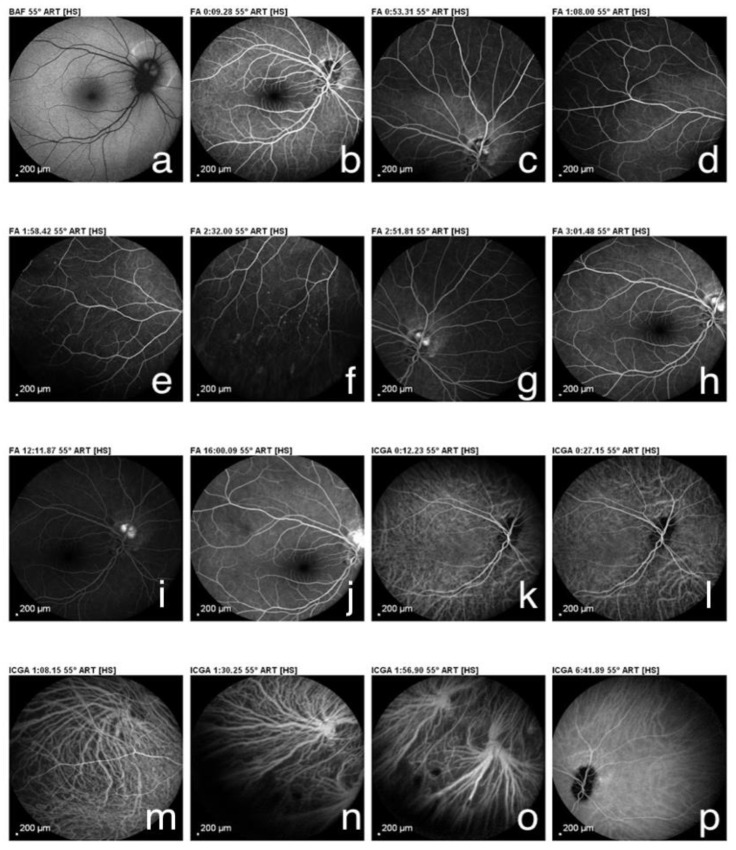
(**a**) Fundus autofluorescence showed a weakly hyperautoflorescent appearance of the peripapillary lesion in the right eye, surrounded by a hyperautoflorescent ring. (**b**–**j**) Fluorescein angiography showed fluorescence of the lesion in the early phase that remained stable in the late phase. (**k**–**p**) Indocyanine green angiography showed an hypocianescent area.

**Figure 3:**
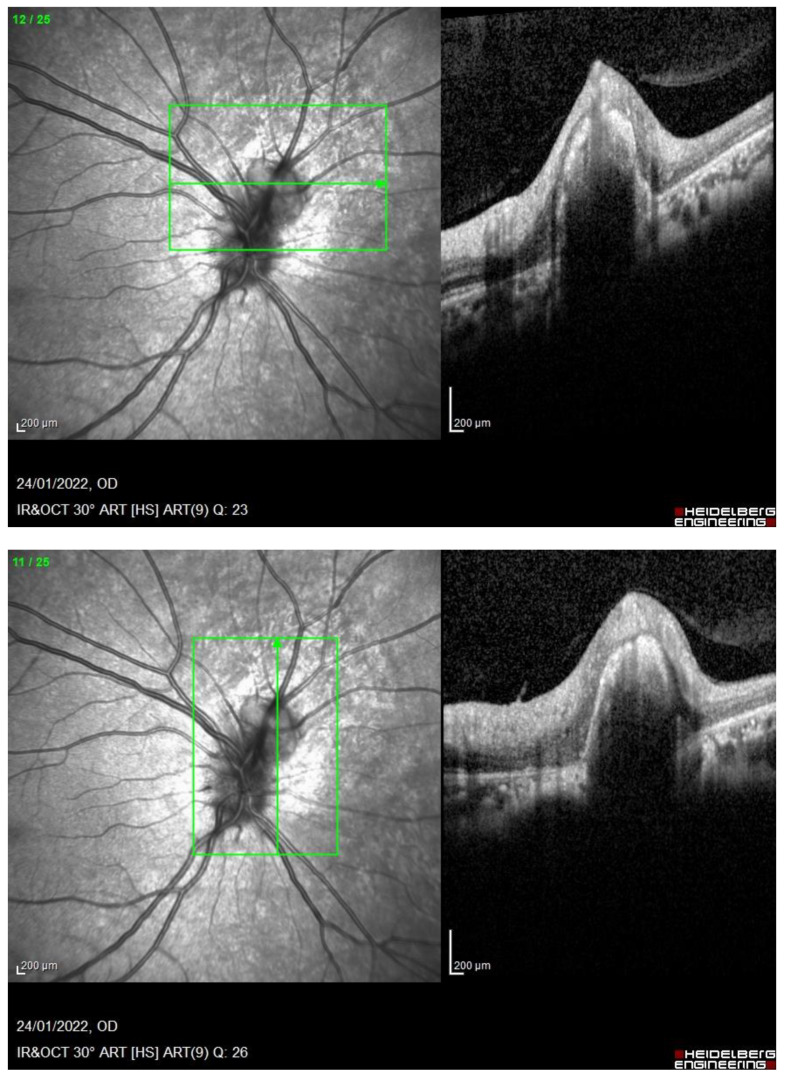

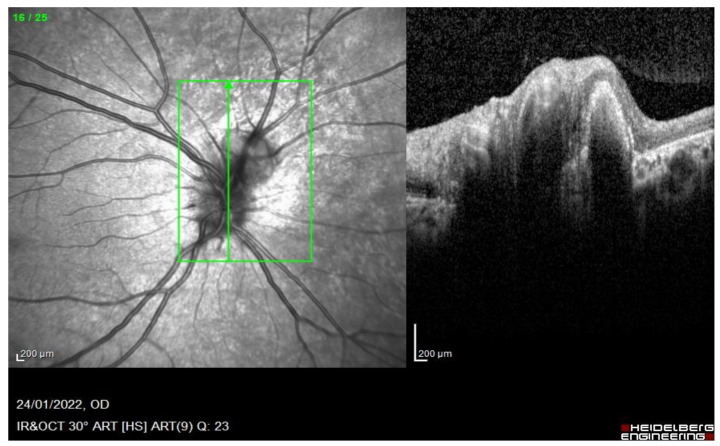
RE. OCT shows a juxtapapillary area of “dome-shaped” retina elevation with hyperreflectivity of the deep retinal layers and shadow cone underneath the retina elevation.

**Figure 4:**
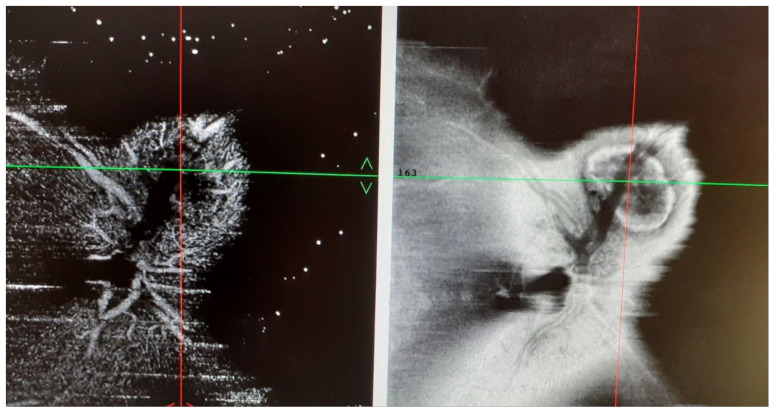

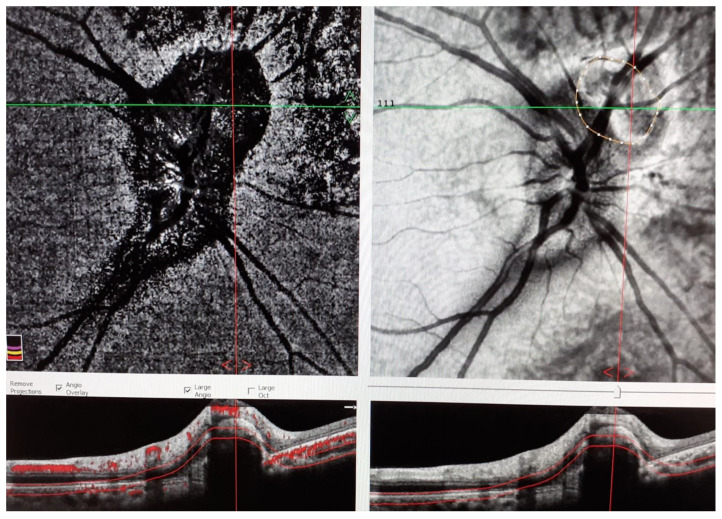
RE.OCT-A shows an oval area of flow void surrounded by normal flow corresponding to the juxtapapillary retina elevation shown by the OCT.

In May 1997, an 8-year-old female patient was admitted to the Uveitis Service of the Department of Ophthalmology of Sapienza University in Rome due to bilateral anterior uveitis diagnosed two months before. Uveitis presented acutely with redness, pain, and decreased visual acuity affecting the left eye (LE) seven days before the right eye (RE). Best-corrected visual acuity (BCVA) was 20/25 in both eyes (BE), and her intraocular pressure (IOP) was 16 mmHg in BE. Perikeratic hyperemia, inferior keratic precipitates, 4+ anterior chamber cells, 2+ flares, and posterior synechiae were observed in BE. Dilated fundus examination revealed vitreous 2+ cells, edema, and hyperemia of the optic disc. Oral steroid treatment (prednisone 1 mg/kg/day) and topical therapy with dexamethasone and mydriatic drops were prescribed. The medical history showed that in August 1995, nineteen months before ocular symptoms, the patient was admitted to a hospital for acute interstitial tubular nephritis (TIN) with renal failure approximately two months after starting oral antibiotic therapy for respiratory disorders. At that time, the girl, with congenital solitary kidney from birth, was admitted to the nephrology department showing vomit, inappetence, loss of weight of approximately 3 kg, and fever. Laboratory investigations revealed glycosuria, proteinuria, cylindruria, anemia (Hb 9.9) increase in creatinine (1.7%) and creatinine clearance 28 mL/min/1,73 m^2^, ESR 67 mm/h, microhematuria, aminoaciduria, and reduced tubular phosphorus. Renal ultrasound only showed the presence of the right hypertrophic kidney and enhanced cortical echogenicity, while renal biopsy was considered unnecessary. Oral prednisone (1 mg/kg/day) was then prescribed by a nephrologist for three weeks, followed by a gradually reduced dose for four weeks. At the first examination in October of the same year, the laboratory analyzed a resumption of renal function with creatinine 0.77 mg /dL and proteinuria 0.3 g/day, and the therapy was stopped. Then, she was submitted to laboratory investigations and other causes of uveitis were ruled out. On day ten, ocular examination BCVA reached 20/20 in both eyes, anterior inflammatory signs decreased significantly, and after one month of therapy, posterior uveitis also improved. A further ophthalmological examination at the beginning of 1999 showed an optic disc with shaded margins, vitreous exudates and a white-grayish area found near the upper papillary margin in the RE, while LE uveitis was inactive. Fluorescein angiography (FA) showed a juxtapapillary focal hyperfluorescent area with leakage in the RE, resembling an active juxtapapillary CNV. Recurrences of bilateral anterior uveitis continued until 2001, and at that time, neither sign of posterior uveitis was detected, showing an inactive juxtapapillary lesion in the RE. The patient was lost from follow-up for many years, and she came back to further control only in January 2022, when she was 32 years old. At fundus examination, a yellowish juxtapapillary superonasal oval shape lesion was observed in the RE. (Figure 1) Fundus autofluorescence (FAF) showed a weakly hyperautofluorescent appearance of the peripapillary lesion in the RE, surrounded by a hyperautofluorescent ring. FA was repeated and showed fluorescence of the lesion in the early phase that remained stable in the late phase. At the indocyanine green angiography (ICGA) examination, the same lesion appeared hypocianescent (Figure 2). Optical coherence tomography (OCT) (Figure 3) and OCT angiography (OCT-A) (Figure 4) were also performed by Heidelberg Engineering. The OCT showed a juxtapapillary area of “dome-shaped” retina elevation with retinal pigment epithelium detachment, hyperreflectivity of the deep retinal layers, and shadow cone underneath the retina elevation. The OCT-A revealed an oval area of flow void surrounded by normal flow corresponding to the juxtapapillary retinal elevation shown by the OCT. FA, ICGA, OCT, and OCT-A findings could be consistent with the final scarring of a juxtapapillary CNV. Since the nephrological checks carried out in the years immediately following the first episode, no new condition of impairment of renal function has been detected.

## 3. Discussion

We described a patient affected by definite TINU syndrome according to the Mandeville classification of 2001 [8], although the timing of the onset of ocular symptoms after the diagnosis of nephritis in our patient was 19 months and not up to 14 as reported [8]. However, the absence of other causes in the diagnostic framework for uveitis, female gender, and her young age, together with the knowledge that the diagnosis of tubulointerstitial nephritis may be delayed often due to nonspecific symptoms, let us hypothesize that our patient was suffering from TINU. Tubulointerstitial nephritis with subsequent acute renal failure was diagnosed during childhood by general symptoms of malaise, anorexia, and asthenia, and through laboratory alterations such as increased creatininemia, proteinuria, and glycosuria approximately two months after the use of antibiotics for a flu illness; therefore, it was probably drug-related. In this case, as already reported in the literature, a rapid improvement in renal function within a few days after the start of systemic steroids, followed by a gradually reduced dose, was observed [11,12,13]. Uveitis in TINU was described as typically anterior mainly in older literature in the 1980s and 1990s, manifesting with cells in the anterior chamber, flares, conjunctival injection, keratic precipitates, and dry eye [1,8]. More recently [14,15], posterior uveitis was frequently found, resulting in inflammatory cells in the vitreous, chorioretinitis, intraretinal hemorrhages, cotton exudates, alteration of retinal vessels, and retinal edema [8,15]. In our case, bilateral uveitis presented as anterior and acute at the onset but became diffused with vitreous cells, posterior uveitis, peripheral vasculitis, and uveo-papillitis in a short time. At that time in 1999, at the age of 10, some years following the diagnosis of TINU, she underwent FA which revealed a peripapillary active juxtapapillary lesion in the RE resembling a CNV. It is noteworthy that in many cases, steroids are ineffective in CNV treatment. Other therapies may be necessary, including the use of anti-vascular endothelium growth factor (VEGF) antibodies. However, at that time, the intravitreal use of anti-VEGF was not considered, since the diagnosis was made before the anti-VEGF era. During the follow-up, the patient was treated with topical and oral steroids, due to their anti-inflammatory properties, until the active disease resolved with a good prognosis, since the patient reached a BCVA of 20/20 in BE. After discontinuation of therapy, a few relapses of mild uveitis were observed that required treatment with short courses of steroids. Twelve years later, at the age of 32, she came back to control without suffering from any noteworthy symptoms and she was submitted to imaging tools such as FAF, FA, OCT, and OCT-A that revealed a juxtapapillary lesion likely compatible with an inactive CNV. This underlines that TINU syndrome, when properly treated, can have a good prognosis.

## 4. Conclusions

Although the majority of cases described in the literature concern anterior not granulomatous uveitis, the most recent studies have shown that several manifestations in the posterior segment have occurred in this syndrome, among which the edema of the optic disc is the most common, followed by macular cystoid edema, chorioretinal lesions, subfoveal CNV, and retinal vasculitis [15]. In the presence of papillitis, optic nerve edema, and juxtapapillary lesion, as observed in our patient, a detailed imaging tools panel was performed to rule out CNV both at the time of the acute phase and during the follow-up, because the CNV development may be also a complication of the later stages of TINU [10]. In our patient, imaging diagnostic tools such as FA, ICGA, OCT, and OCTA were performed, permitting us to monitor the juxtapapillary CNV during 25 years of follow-up. Uveitis and CNV were restored after oral steroid therapy, and the visual prognosis was good, with BCVA 20/20 in both eyes. As far as our knowledge, this is the first case of juxtapapillary CNV with papillitis associated with TINU syndrome. An early diagnosis should be suggested, because CNV could need a more intensive treatment with anti-inflammatory steroidal or immunosuppressive therapies other than with antiproliferative drugs [10].

## Data Availability

Data are contained within the article.
